# Endogenous pain modulation after sleep restriction in migraine: a blinded crossover study

**DOI:** 10.1186/s10194-024-01879-z

**Published:** 2024-10-03

**Authors:** Jan Petter Neverdahl, Martin Uglem, Dagfinn Matre, Kristian Bernhard Nilsen, Knut Hagen, Gøril Bruvik Gravdahl, Trond Sand, Petter Moe Omland

**Affiliations:** 1 Department of Neuromedicine and Movement Sciences, Faculty of Medicine and Health Sciences, NTNU, Norwegian University of Science and Technology, Trondheim, 7491 Norway; 2 NorHEAD - Norwegian Centre for Headache Research, NTNU, Trondheim, Norway; 3 Section for Clinical Psychosis Research, Department of Research and Innovation, Division of Mental Health and Addiction, Oslo University Hospital, Oslo, Norway; 4 Department of Neurology and Clinical Neurophysiology, St. Olavs hospital, Trondheim University Hospital, Trondheim, Norway; 5 National Institute of Occupational Health, Oslo, Norway; 6 Section for Clinical Neurophysiology, Department of Neurology, Oslo University Hospital, Oslo, Norway; 7 Clinical Research Unit, St. Olavs Hospital, Trondheim University Hospital, Trondheim, Norway; 8 Department of Radiology and Nuclear Medicine, St. Olavs Hospital, Trondheim University Hospital, Trondheim, Norway

**Keywords:** Temporal summation, Conditioned pain modulation, Adaptation, Interictal migraine, Insufficient sleep, Sleep-related migraine

## Abstract

**Background:**

Patients with migraine are vulnerable to insufficient sleep, but the impact of sleep restriction is largely unknown. In addition, the importance of sleep may be different in patients with migraine who mostly have attack onsets during sleep, so called sleep-related migraine, compared to patients with non-sleep-related migraine. In this study we investigate the effect of sleep restriction on endogenous pain modulation in patients with migraine and healthy controls. We also compared the effect of sleep restriction in sleep-related and in non-sleep-related migraine.

**Methods:**

Measurements were conducted in 39 patients with migraine between attacks and 31 controls, once after habitual sleep and once after two consecutive nights of partial sleep restriction. There were 29 and 10 patients with non-sleep-related and sleep-related migraine respectively. Test stimulus was 2-min tonic noxious heat to the left volar forearm. Temporal summation was calculated as the regression coefficient for rated pain in the late part of this 2-min stimulation. Conditioning stimulus was right hand-immersion in 7 °C water. Conditioned pain modulation was defined as the difference in rated pain with and without the conditioning stimulus and was calculated for temporal summation and mean rated pain for the test stimulus. The effect of sleep restriction on temporal summation and conditioned pain modulation was compared in migraine subjects and controls using two-level models with recordings nested in subjects.

**Results:**

Conditioned pain modulation for temporal summation of heat pain tended to be reduced after sleep restriction in patients with migraine compared to controls (*p* = 0.060) and, in an exploratory analysis, was reduced more after sleep restriction in sleep-related than in non-sleep-related migraine (*p* = 0.017). No other differences between groups after sleep restriction were found for temporal summation or conditioned pain modulation.

**Conclusion:**

Patients with migraine may have a subtly altered endogenous pain modulation system. Sleep restriction may have an increased pronociceptive effect on this system, suggesting a mechanism for vulnerability to insufficient sleep in migraine. This effect seems to be larger in sleep-related migraine than in non-sleep-related migraine.

**Supplementary Information:**

The online version contains supplementary material available at 10.1186/s10194-024-01879-z.

## Background

The importance of sleep for migraine is well-known among clinicians and patients. Migraine attacks can be triggered by short-term disturbed sleep [1], while sufficient sleep can protect against migraine attacks [2]. In addition, insomnia is known to increase the risk of developing migraine [3]. However, the connection between sleep and migraine is largely unexplained [4].

Experimental sleep restriction increases pain sensitivity in healthy subjects [5]. It is also a promising model for investigating the effect of insufficient sleep on migraine [6–9]. Patients with migraine have increased pain perception during [10], and between attacks [11], which may be caused by altered endogenous pain modulation [12]. Conditioned pain modulation (CPM) is the human equivalent of diffuse noxious inhibitory control (DNIC) originally described by Le Bars et al. [13], where a noxious stimulus is transmitted from the dorsal horn to the caudal medulla, resulting in diffuse peripheral inhibition via the dorsolateral funiculi [14]. In CPM, one noxious stimulus (conditioning stimulus; CS) reduces the painfulness of another simultaneous noxious stimulus (test stimulus; TS) [13, 15]. CPM is thought to represent net effect of descending pain pathways, activated by bottom-up-mechanisms [16]. Although he underlying mechanisms for DNIC and CPM are not fully elucidated [17], intact spinal and medullary structures are necessary for both these effects [18, 19], while supraspinal brain areas may also be involved in CPM [20]. Similarly, serotonergic systems may be involved in DNIC [21], while dopaminergic neurotransmission may also be involved in CPM [22]. In temporal summation of pain, increasing pain in the presence of constant tonic or repetitive (≥ 0.33 Hz) stimuli, is considered the human correlate of wind-up [23]. Although temporal summation is not equivalent to central sensitisation [23], they share many attributes, and temporal summation is therefore used as a proxy for central sensitisation [24–26].

Decreased CPM and/or increased temporal summation in patients with migraine between attacks has been found by some [27–32], but not all studies [28, 33–37]. Alterations in pain processing in migraine between attacks may therefore be subtle [38]. Additionally, pain processing may vary within the migraine cycle [39–42], or be specific to subgroups of migraine. For instance, patients with migraine with mostly attack onsets during sleep, so called sleep-related migraine, seem to differ from patients with non-sleep-related migraine for several objective measures of sleep quality [43], and for the effects of sleep restriction on neurophysiological measures [7, 9].

In this blinded crossover study, we investigate the impact of insufficient sleep on migraine. We compare the effect of sleep restriction on temporal summation of heat and mechanical pain and CPM in patients with migraine between attacks and controls. Sleep restriction may affect an already subtly altered pain modulation system in patients with migraine differently than in healthy controls. We therefore hypothesise that sleep restriction will have a more pronounced pro-nociceptive effect in patients with migraine compared to controls. In an additional exploratory analysis, we compare the effect of sleep restriction in sleep-related and non-sleep-related migraine.

## Methods

### Design

**Fig. 1:**
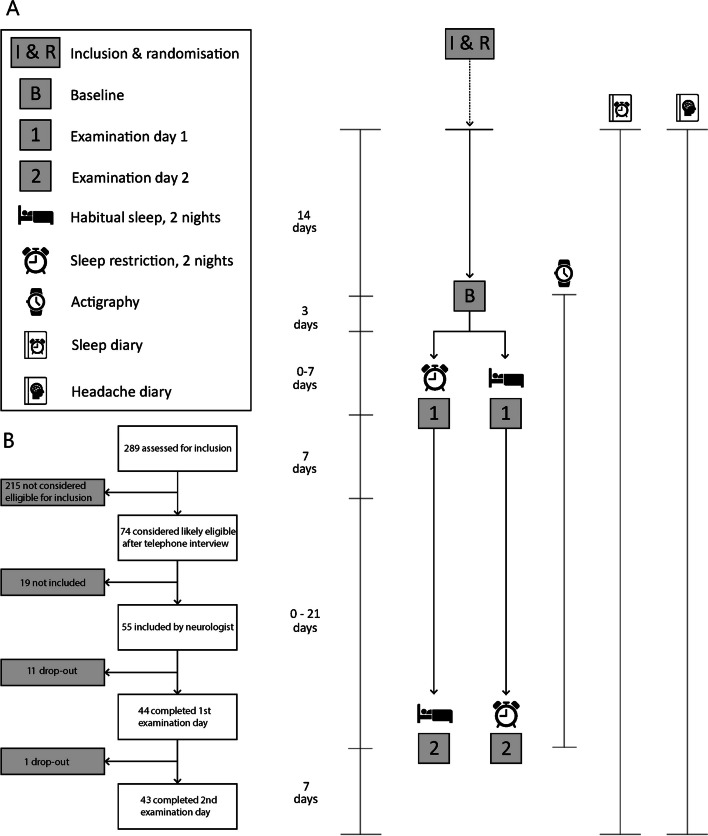
Study overview. **A** Participants completed a sleep diary and wore an actigraph in the indicated time period. Patients also completed headache diaries. For the sleep restriction condition, participants slept four hours for two consecutive nights preceding one of the examination days. We balanced and randomised order of sleep conditions between examination days. To ensure flexibility, the interval between baseline and Day 1, and Day 1 and Day 2, was allowed to vary between 3–10 days and 1–4 weeks, respectively. **B** Overview of the inclusion process for patients with migraine

The data presented in this study stems from a larger data collection conducted from May to December 2016, including heat and pain pressure thresholds that are published in Neverdahl et al. 2022 [9]. In the present blinded cross-over study participants came to our lab for identical procedures at baseline testing and subsequently at two examination days (Day 1 and Day 2), once after habitual sleep and once after sleep restriction (Fig. 1). The purpose of the baseline testing was to minimise learning and order effects in subsequent examination days. No data was collected during baseline testing. For the sleep restriction condition participants were instructed to sleep four hours for two consecutive nights. Participants were instructed to avoid daytime napping. The same investigator tested all participants. The examiner was blinded to diagnosis and sleep condition during data collection and data analysis. To ensure and maintain blinding, a study nurse handled the logistics of the study, and collected headache and sleep diaries and questionnaires. The examiner performing the laboratory examinations did not participate in recruitment, inclusion or exclusion of participants before data collection or have knowledge of diagnosis or sleep condition until after the data from all subjects was collected, processed, and exclusions due to preset criteria were made. Participants were reminded to not disclose diagnosis or sleep condition at the start of each meeting. We randomised order of sleep condition between examination days. To ensure that the order of sleep condition was balanced in controls and migraine subjects, we used separate block randomisation for migraine patients and controls. To ensure flexibility, the interval between baseline and Day 1, and between Day 1 and Day 2, was allowed to vary between 3–10 days and 1–4 weeks, respectively. We set a lower limit of one week between Day 1 and Day 2 to avoid potential residual sleep restriction effects.

### Test subjects

Patients with migraine and controls were recruited through intranet advertisement at the Norwegian University of Science and Technology and St. Olavs Hospital, Trondheim University hospital, and screened by a study nurse according to predetermined exclusion criteria (Table 1). The study nurse ensured that migraine patients and controls were matched for age and sex. Patients with probable migraine were later evaluated by neurologists in accordance with the beta version of the third edition of the International Classification of Headache Disorders (ICHD-III beta) [44]. These neurologists did not participant in the laboratory examinations, and the evaluation by neurologist occurred prior to the baseline day. Patients were included if they had episodic migraine, and 1–6 migraine attacks per month. Migraine patients were allowed to use symptomatic migraine treatment during the study period, while prophylactic migraine treatment was not permitted within 4 weeks before and during the study period. Controls were allowed to report minor headache less than once per month. Controls with occasional headache were asked if they had consulted a physician regarding headache, if the headache was experienced as painful, i.e. having a more than mild intensity, and if they used abortive medication for their headache. They were not included if they confirmed more than one of these three questions (Table 1).

**Table 1:** Exclusion criteria for all participants

Age range 18–65 Co-existing tension type headache (≥ 7 attacks/month for patients with migraine)
Neurological or psychiatric disorder with decreased function
Confirmed sleep disorder
Infectious disease
Connective tissue disorders
Metabolic, endocrine, or neuromuscular disease
Acute or chronic pain disease
Recent injury affecting function
Neoplastic disease
Previous craniotomy or cervical neurosurgery
Pregnancy
Cerebrovascular or symptomatic heart disease
Pulmonary disease
Hypertension (> 160/110)
Breastfeeding
Medication for acute or chronic pain
Neuroleptic or anti-epileptic drugs
Anti-depressive drugs
Cardiovascular, pulmonary, or antihypertensive drugs
Other drugs that might influence neuronal, vascular, or muscular function
Body mass index (BMI) < 17 or > 35
Alcohol or drug abuse
Ferromagnetic implants
Prophylactic allergy treatment
Additional exclusion criteria for controls
≥ 1 minor headache per month
When occasional headaches, controls were not included if ≥ 1 of the following were affirmed:
Consultation by a physician
The headache was experienced as painful
The headache caused use of abortive medication

Forty-four patients with migraine and 31 healthy, sex- and age-matched controls were included in the experimental procedure (Fig. 1). One patient withdrew consent, and one was excluded because of incomplete headache diary. Incomplete data from one examination day after sleep restriction from another migraine patient was not used in analysis. Migraine patient examinations were classified as interictal if there was no migraine headache in the 24 h preceding or following the visit to our lab, as preictal if they experienced a migraine headache in the following 24 h, as postictal if they experienced a migraine headache in the 24 h preceding the examination day, and as ictal if they experienced a migraine headache during the examination. Three patients did not have interictal recordings.

39 migraine patients had at least one interictal recording and could be included in the analysis. 30 of these migraine patients were assessed in the interictal phase after habitual sleep and 30 of them in the interictal phase after sleep restriction. 21 of the 39 migraine patients had two interictal recordings. As described later, we also performed a secondary sensitivity analysis for significant findings using a 48-h cut-off for the interictal-preictal phase border, in line with the recommendations of Peng et al. [45]. All included healthy controls completed the assessments.

Based on structured interview conducted by a study nurse prior to the baseline day, patients with migraine were divided into subgroups based on clinical features; as sleep-related migraine if migraine attacks typically started “upon waking” or “during the night (waking me up)”, and as non-sleep-related if migraine attacks typically started “during daytime before noon”, “during daytime after noon”, or there was “no regular onset time” Engstrøm et al. [46]. We did not compare patients with migraine with or without aura, as the migraine with aura subgroup was small (*n* = 5).

### Collection of clinical migraine and sleep variables

Participants were instructed by a study nurse and completed sleep diaries. This included registration of any daytime napping. Migraine patients also completed headache diaries about headache onset, duration, intensity, photo- and phono-phobia, use of medication and aura on paper from two weeks preceding baseline until one week after Day 2 (Fig. 1). Karolinska Sleepiness Scale (KSS) (score 1–9) was used to quantify sleepiness at the end of each examination [47]. Participants completed a questionnaire with clinical sleep variables at home, including tendency to fall asleep at daytime quantified by Epworth Sleepiness Scale (ESS, score 0–3 for eight questions, yielding a maximum score of 24 [48]). Insomnia symptoms were quantified by Insomnia Severity Score (ISS, score 0–3 for four questions, yielding a maximum score of 12 [49]). A study nurse collected data on clinical migraine variables in a structured interview, which included years since diagnosis and intensity and frequency of photo- and phono-phobia. Headache diaries were used to quantify hours between test days and the next attack.

Participants wore a wrist actigraph (Actiwatch Spectrum Plus, Philips Respironics, U.S.A), from baseline to Day 2 (Fig. 1). The actigraph recorded total sleep time during the whole data collection. Total sleep time from the two days preceding examination days were averaged. Rest intervals defined by the actigraphy software (Philips Actiware 6, Philips Respironics, U.S.A) were corrected semi-manually in a hierarchal manner [50].

### Experimental procedure

**Fig. 2:**
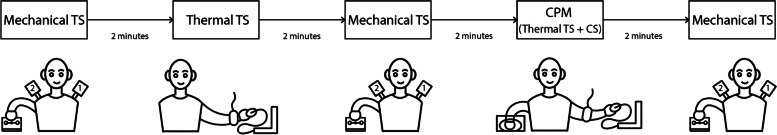
Conditioned pain modulation (CPM) protocol. Firstly, participants underwent mechanical test stimulus (TS), i.e., determination of pressure pain threshold (PPT) and suprathreshold pain level (PP5, at VAS = 5/10 cm); at left (1) and right (2) trapezius muscles. Second, a two-minute tonic thermal TS at «pain6» (VAS = 6/10 cm) intensity was applied to the left volar forearm while participants continuously rated their pain. Third, mechanical TS was repeated. Fourth, thermal TS was repeated simultaneously with immersion of the right hand in circulating water (7 °C) (conditioning stimulus, CS); this constituted the CPM condition. Participants rated CS-induced pain by a verbal NRS. Finally, the mechanical TS was repeated for the third time

Participants abstained from nicotine or caffeine from midnight and arrived either at 08:00 or 09:30 both examination days. To maintain blinding, a study nurse ensured that these starting times for the examinations were distributed similarly between migraine subjects and controls. On both examination days participants successively went through eight parts: 1) a structured interview including questions about caffeine, alcohol, and nicotine use in the preceding 24 h, present hormonal contraception use, and time of last menstruation; 2) self-reported medication use, existence of ongoing headache, and details concerning potential ongoing headache (the researcher was blinded to this information); 3) blood pressure measurement; 4) a psychomotor vigilance test (PVT) to quantify alertness as a correlate to sleep deprivation, a 10-min simple reaction time test with 2–10 s interstimulus intervals and a total of 70–75 stimuli using a custom-written C +  + program from the National Institute of Occupational Health in Norway) [51, 52]; 5) determination of heat pain tolerance threshold (HPTT at verbal numerical rating scale (NRS) equal to 10 where 0 is defined as ‘no pain’ and 10 as ‘worst imaginable pain’ measured three times); 6) determination of the “pain6”-temperature to be used in the CPM protocol, that is the temperature producing pain = 6 on the NRS (Details on determination of pain6 is described in Supplementary material); 7) the CPM protocol, including tonic heat pain for the quantification of temporal summation of pain (Fig. 2); and lastly 8) a self-report questionnaire on headache intensity, character, and sleepiness measured by KSS. KSS was assed at the end of the examination to ensure that differences in sleepiness between sleep conditions also were present at the end of the examinations. To maintain blinding, the participants completed this self-report questionnaire at the end of the session, put this into an envelope and put the closed envelop into a container outside the examination room.

### Conditioned pain modulation

#### Test stimulus (TS)

We used a tonic thermal TS, as well as a secondary mechanical pressure TS [53]. Two minutes of tonic noxious heat at pain6 level was applied to the volar left forearm using a transversally placed hand-held rectangular 25 × 50 mm Peltier element thermode (Somedic Sales AB, Stockholm, Sweden) while participants continuously rated their subjective pain experience on a computerised visual analogue scale (VAS, 0–10 cm, National Institute of Occupational Health, Norway) by scrolling a wheel on a computer mouse. The upper limit for the thermal TS was set at 49 °C. Pain6 was determined each test day and used for both the thermal TS and the CPM condition, as a previous study found increased CPM after sleep restriction, and attributed this to increased painfulness of the TS after sleep restriction [54]. There was no overlap between the location used for determination of pain6 or the two thermal TS to avoid habituation or sensitisation [55].

Pressure stimuli were applied to both trapezius muscles, at sites 1/3 from the posterior edge of the acromion to the C7 as measurements on this site produced more repeatable results in a pilot study by a collaborating group [56]. We used a FDMIX digital hand-held force gauge instrument (Wagner instruments, Greenwich, U.S.A., probe size 1 cm^2^, when force = 10 Newton (N) correspond to a pressure = 100 kPa), and a custom-written program (National Institute of Occupational Health, Norway) to provide real-time visual feedback of force and ensure a steady increment of 50 kilopascal (kPa)/second by the experimenter [56]. Participants continuously indicated subjective pain experience on a hand-operated VAS device (0–10 cm, sampled digitally). The VAS device indicated the pressure pain threshold (PPT), and stimulation was ended at VAS = 5/10 cm or Force = 100 N. The applied force at VAS = 5/10 cm was defined as “PP5” (suprathreshold pressure pain). Hence, PPT and PP5 were measured in the same procedure.

#### Conditioning stimulus (CS)

The CS consisted of two minutes of immersion of the right hand in 7 °C circulating water [54] (Lab Companion RW-0525G, Biotechnical Services Inc, U.S.A). The hand was immersed to the wrist, keeping the fingers spread. The water was circulating to ensure laminar flow, consequently avoiding local heating of the water directly adjacent to the skin. In cases where the CS was aborted before two minutes, the thermal TS and corresponding VAS scoring was continued as planned, unless patients subsequently aborted the thermal TS. After the two minutes participants verbally rated the overall painfulness of the CS using verbal NRS [55]. For the CPM protocol, conditioned thermal TS was delivered in parallel with CS, while conditioned mechanical TS was delivered two minutes after CS (Fig. 2).

### Data analysis

PVT reaction times were inverted (1/second), and the 10% smallest and largest values were removed for each subject and session [51]

#### Thermal stimulation and pain measures (VAS)

Measurements were handled with an intention to treat approach, i.e., included although 50% sleep restriction was not attained, mean VAS below two or above eight was not attained for the thermal TS, or in cases of abortion of the CS. Three and 29 out of 122 tonic thermal stimuli had a mean VAS below two or above eight, respectively. Forty out of 122 CS were aborted by the participants due to intolerable pain.

**Fig. 3:**
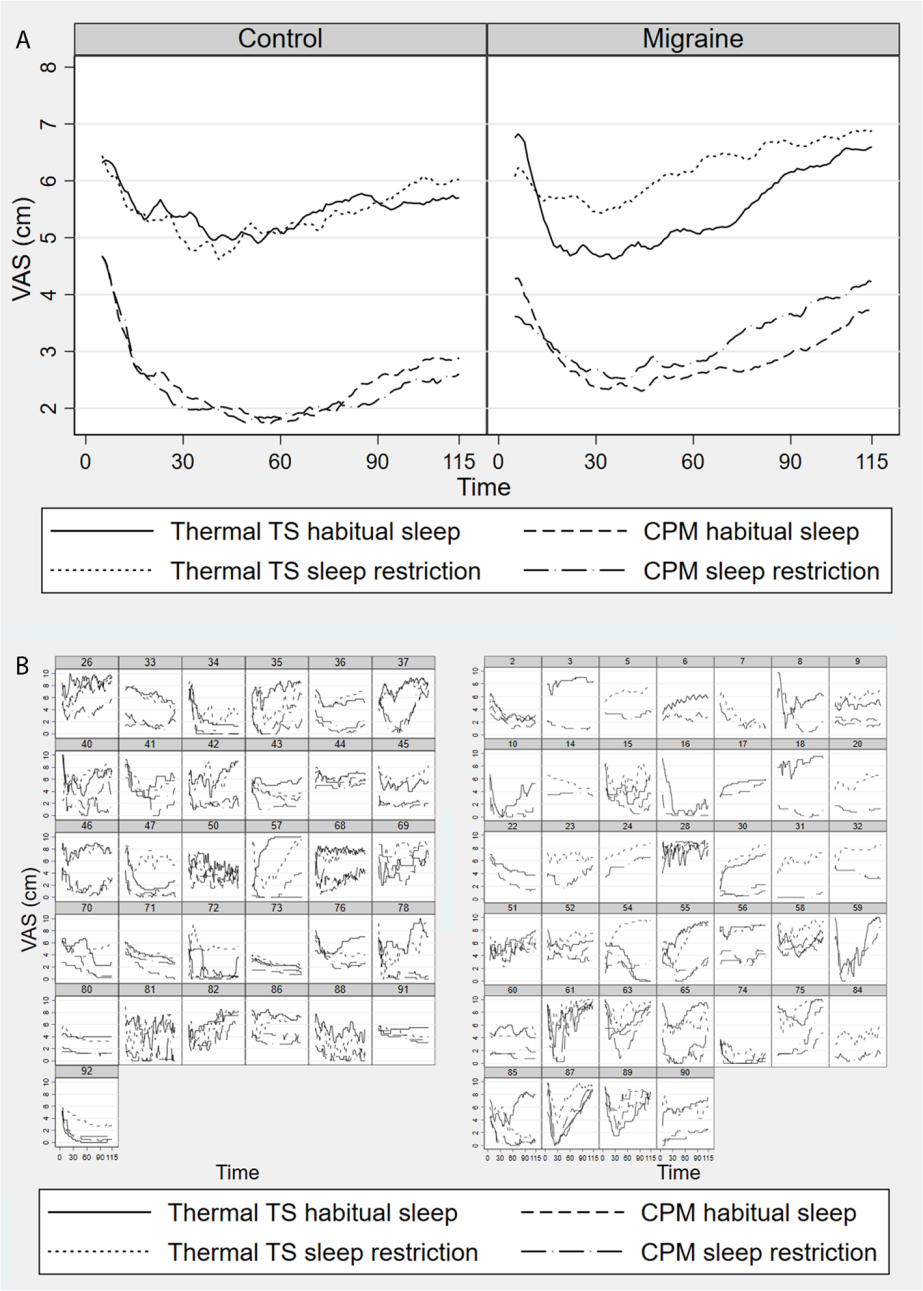
Grand means and individual plots from the conditioned pain modulation (CPM) protocol. Visual analogue scale (0–10 cm). TS: test stimulus. CPM: Conditioned pain modulation. Y-axis unit is VAS-pain, x-axis unit is time in seconds. **A** Grand means for VAS responses to the thermal test stimulus (TS) from the thermal part of the conditioned pain modulation (CPM) protocol. Each line shows VAS per time for combinations of group (patients with migraine vs controls), sleep condition (sleep restriction vs habitual sleep), and stimulus condition (CPM vs thermal TS). The VAS responses follow a typical pattern for noxious tonic heat stimulations, including an initial peak, subsequent adaptation with a nadir around 25–65 s, and final temporal summation. The first and last five seconds were removed due to varying VAS measurements in these time periods. **B** Same as in A, showing VAS measurements from each test subject (numbered) for combinations of sleep and stimulus condition. Controls to the left and patients with migraine to the right

**Fig. 4:**
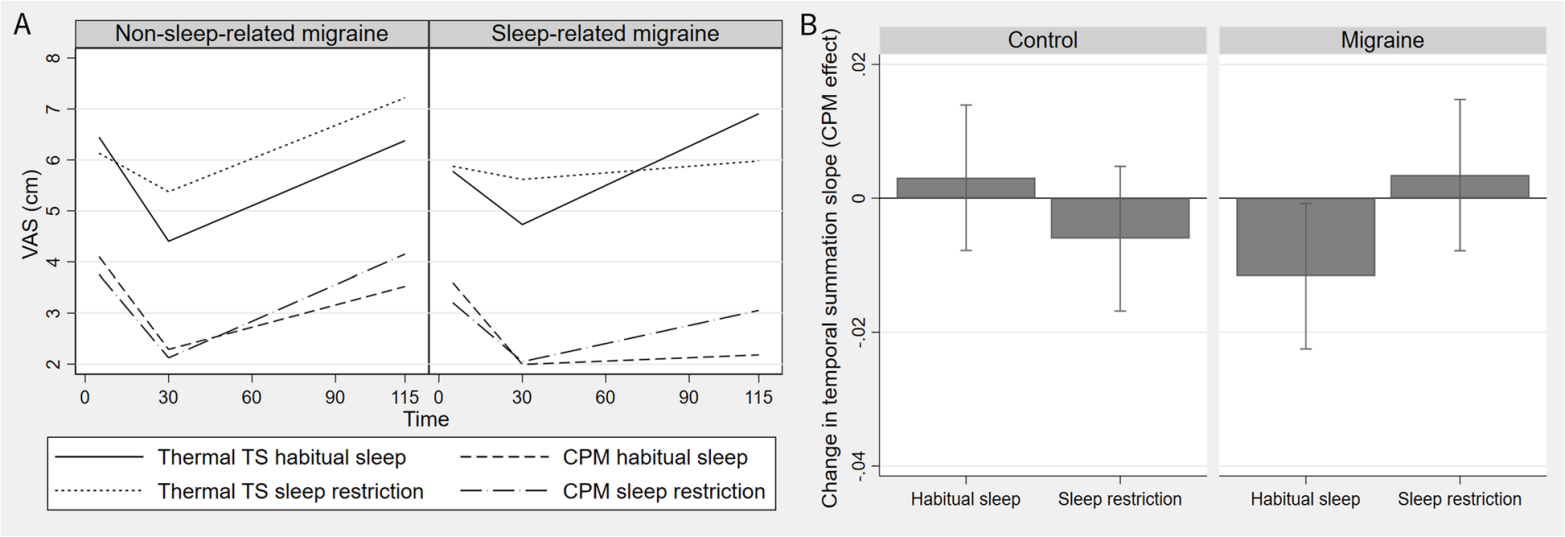
Piecewise model and contrasts from the conditioned pain modulation (CPM) protocol. VAS: Visual analogue scale (0–10 cm). TS: test stimulus. CPM: Conditioned pain modulation. Time in seconds. A) Y-axis unit is VAS pain, x-axis unit is time in seconds. Linear regression lines from the piecewise regression model for the thermal part of the CPM protocol. Each regression line shows VAS by time for each combination of group (patients with migraine vs controls, sleep condition (sleep restriction vs habitual sleep), and stimulus condition (CPM vs thermal test stimulus (TS)). There was a significant three-way interaction between group, sleep, and stimulus condition. B) Bar plot showing CPM effect on temporal summation of pain for patients with migraine and controls after habitual sleep and sleep restriction; the bars show change in slope of the temporal summation regression line (ratio of vas/cm) from 30 s with only TS and TS in addition to CS (CPM condition). CPM effect on temporal summation of pain tended to be decreased after sleep restriction in patients with migraine compared to controls. In the column for patients with migraine in A, this can be seen as diverging regression lines for thermal TS and CPM after habitual sleep, while the regression lines converge after sleep restriction. There was also a tendency toward increased CPM effect on temporal summation of pain in patients with migraine compared to controls after habitual sleep. This can be seen in A as slightly converging regression lines for the thermal TS and CPM conditions after habitual sleep in controls, while the corresponding regression lines diverges in the migraine group

We frequently observed an initial peak in VAS score at 5–10 s, subsequent adaptation to a nadir around 25–65 s, and final temporal summation (Fig. 3). A regression coefficient for VAS by time for the early part of stimulation was used as a measure of adaptation while the coefficient from the late part of the stimulation was used as a measure of temporal summation. To determine the point of separation between the early and late part, to be used in calculations, the Akaike and Bayesian information criterion indicated optimal knot placement at 32 s (Fig. 4A). To avoid over-specification, we chose time knot placement at 30 s for the final analysis. The first and last five seconds were removed due to varying VAS.

#### Pressure stimuli

Eighty-six out of 488 measurements exceeded the pre-set limit of 100 N. For this reason, we used *estimated* PP5 for suprathreshold pressure pain. Estimated PP5 was calculated for each measurement using a linear regression model between force and VAS ratings. *R*^2^ < 0.80 indicated lack of linearity and resulted in exclusion of PP5 [56], and ten PP5 measurements from three controls were excluded.

### Statistics

#### Primary analysis

We ran separate multilevel models for temporal summation of pain and for CPM-effects on temporal summation, and thermal mean VAS (main variables). CPM effect was defined as the difference in pain between the TS and the CS. For completeness, we also ran separate multilevel models on secondary variables, i.e. adaptation slopes and for CPM-effects on adaptation, PPT, PP5, and initial peak pain (at five seconds). The models were specified as two-level models with recordings nested within subjects. The interaction terms including group and sleep condition were considered the main statistical outcome measures in accordance with the aims of our study.

The fixed parts of all models were defined à priori; in the primary analyses this included main effects of group (patients with migraine vs controls), sleep condition (sleep restriction vs habitual sleep) and stimulus condition ((CS + TS vs TS, i.e. CPM effect)), and their respective two-way and three-way interactions. The piecewise regression model for thermal stimuli also included main effects of time (5–30 s – adaptation; 30–115 s – temporal summation), and interactions between time and group, time and sleep condition, and time and stimulus condition. Random coefficients and covariance matrices were included based on likelihood ratio tests. Details on model specifications in the primary analysis can be found in the Supplementary material, and in Supplementary Table S2. Normality of level-one residuals and higher-level random effects was checked visually by histograms and qq-plots, and response variables were transformed when deemed necessary, that is for PPT (natural logarithm of VAS) and PP5 (VAS^−0.2^).

Mean VAS for the thermal TS for each combination of group (patients with migraine vs controls), sleep condition, and stimulus condition (CPM vs. thermal TS) were calculated and used in a separate CPM analysis [56].

All primary analyses followed the same structure, with 1) testing for significant main effect (for temporal summation, adaptation, or CPM) for both groups and both sleep conditions combined; 2) testing for significant two-way interaction between group and sleep condition (main outcomes). Stimulus condition was included in CPM analyses (three-way interaction). A significant three-way interaction prompted step 3) where lower-level interactions in each group and sleep condition were tested for significance. Step 1) was conducted to assess whether our protocol was sufficient to produce adaptation, temporal summation, and CPM. We chose testing main effects in combined groups to ensure sufficient power. Comparisons between groups was done after significant three-way interactions to reduce the number of tests. To aid the interpretation of a significant three-way interaction without too many sub-analyses, only a subset of available comparisons between variables was done based on predefined theoretically interesting contrasts. We did not correct for multiple testing, due to the exploratory nature of these analyses and to avoid likelihood of type II errors [57]. All analyses were performed with STATA version 17.0 (StataCorp LLC).

#### Secondary analyses

In exploratory analyses we specified a separate multilevel model using sleep-related or non-sleep-related migraine as the group factor; model specifications for exploratory analyses can be found in the Supplementary material and in Supplementary Table S4. Due to the sleep-related migraine group being small (*n* = 10; 6 after habitual sleep, 7 after sleep restriction), we used the Kenward-Roger correction for small sample inference [58]. Additionally, we repeated analyses for CPM effect on temporal summation using a 48-h cut-off for the interictal-preictal phase border as this was the only analysis producing a significant result. This sensitivity analysis was only performed for main group comparison between interictal migraine and controls, and not between sleep-related and non-sleep-related migraine. The decision to repeat analyses on variables yielding significant results in the primary analysis was done à priori. See Supplementary Table S3 for details about participants included in these analyses.

#### Sample size and power calculation

Forty-four patients with migraine and 30 controls in a cross-sectional design yields groups of similar size after excluding non-interictal examination days in our experience, and we expected approximately 20 migraine patients to have interictal recordings both after habitual sleep and sleep restriction. 30 subjects in each group in a two-sample t-test yields approximately 70% power to detect a low medium-sized effect (0.65 standard deviations (SD)). 20 patients with migraine with two interictal examination days in a paired t-test yields a power of 79% to detect the same effect size (0.65 SD). Results with *p*-values < 0.05 were considered significant. Results with *p*-values between 0.05 and 0.10 were discussed when appropriate [59, 60]. In the cases where one examination day was excluded, the other examination day was still included in the analyses, as multilevel models adequately handle missing data [61].

## Results

### Demographic data

Controls and migraine patients were similar in demographic data. Patients with sleep- and non-sleep-related migraine also had similar demographic data, but patients with sleep-related migraine were slightly older and had fewer oral contraceptive users than non-sleep-related migraine (Table 2).

**Table 2:** Demographic and clinical data after exclusions

	**Controls**	**Interictal migraine**	**Sleep-related migraine**	**Non-sleep-related migraine**
Total number of subjects	31	39^a^	10	29
Age	36.2 (10.6)	39.2 (9.1)	42.7 (11.8)	36.7 (8.0)
Age range	20–56	20–60	32–60	20–48
Body mass index	24.4 (3.4)	24.3 (3.9)	24.5 (3.6)	24.3 (3.9)
Women/Men	23/8	32/7	9/1	23/6
MwoA/MwoA + MA/MA	NA	22/12/5	6/3/1	16/9/4
Days from last menstruation^b^				
Before habitual sleep	17.7 (9.2)	16.8 (7.8)	17.5 (5.5)	18.3 (15.0)
Before sleep restriction	14.9 (9.6)	15.8 (11.0)	13.2 (6.9)	16.8 (12.2)
Use of hormonal contraception (n)	9	13	2	11
Menopause	4	5	2	3
Epworth Sleepiness Scale (0–24)	6.7 (4.0)	6.7 (3.8)	6.2 (3.8)	6.9 (3.8)
Insomnia Severity Score (0–12)	3.6 (1.9)	4.7 (2.7)	4.8 (2.7)	4.7 (2.7)
Years with headache	NA	22.0 (10.2)	24.9 (9.0)	21.0 (10.6)
Migraine days/month^c^	NA	4.8 (2.9)	5.0 (2.4)	4.8 (3.0)
Migraine intensity (1–4)^d^	NA	2.8 (0.6)	2.7 (0.8)	2.8 (0.6)
Headache duration in hours^e^	NA	10.0 (14.7)	6.5 (4.5)	11.3 (16.7)

Data displayed as mean (SD), range, or number (n). MwoA: Migraine without aura. MA + MwoA: Attacks with and without aura (both diagnoses according to ICHD-III (beta) criteria). MA: Migraine with aura (in 100% of attacks). NA: Not applicable. SM: Sleep-related migraine (headache start “upon waking” or “during the night (waking me up)”. Non-sleep-related migraine: headache start “during daytime before noon”, “during daytime after noon”, or “no regular onset time” Demographic and clinical data in controls, patients with migraine with one or more interictal test days, and the subgroup of patients with migraine with two interictal test days. ^a^60 examination days from 39 patients with migraine with at least one recording in the interictal phase using a 24-h cut-off for the interictal-preictal phase border. See Supplementary Table S3 for information on patients with migraine using a 48-h cut-off for the interictal-preictal phase border. ^b^Days from last menstruation are calculated without data from 4 examinations in migraine patients and 4 examinations in controls that had ≥ 85 days since their last menstruation because of continuous use of contraceptives, and without menopausal subjects. ^c^Days with a migraine headache per month the last 3 months. ^d^Intensity of migraine headache during attacks: 1: mild, 2: moderate, 3: severe, 4: extreme. ^e^Average duration of headache with or without use of medication

Participants in both groups slept close to the goal of four hours during sleep restriction. Sleep time during sleep restriction was approximately 56% of sleep time during habitual sleep. Sleepiness was increased similarly in both groups after sleep restriction (Table 3).

**Table 3:** Selected sleep variables by group and sleep condition. Mean (SD) or counts

	**Controls (*N* = 31, 62 recordings)**		**Patients with migraine (*N* = 39, 60 interictal recordings)**	
< 2 h between HS and SR (n)	2		4	
Sleep time ratio (SR/HS, %)	56 (6.4)		56 (15.2)	
	**Habitual sleep**	**Sleep restriction**	**Habitual sleep**	**Sleep restriction**
Number of recordings (n)	31	31	30	30
Total sleep time (hours)^a^	7.0 (0.6)	3.9 (0.3)	6.7 (1.2)	3.7 (0.9)
Time in bed (hours)^b^	7.8 (1.0)	5.3 (1.4)	7.0 (1.3)	5.4 (1.3)
Karolinska Sleepiness Scale (1–9)^c^	1.8 (1.5)	4.5 (2.6)	2.4 (1.9)	5.1 (2.4)
Psychomotor vigilance test (1/s)^d^	3.1 (0.2)	3.1 (0.2)	3.1 (0.3)	3.0 (0.3)

*N* = number of test subjects; in patients with migraine with one or more interictal test days, either one after habitual sleep or SR, or both. *HS* Habitual sleep. *SR* Sleep restriction. ^a^Total sleep time from the two nights preceding each test day, collected by actigraphy. ^b^Time in bed was extracted from sleep diaries. ^c^Karolinska Sleepiness Scale (1–9), measured after each test day. ^d^Psychomotor vigilance test (PVT), mean reaction times were inverted (1/s)

Patients with migraine aborted CS more often than controls (12 vs 10 after habitual sleep, 14 vs 4 after sleep restriction, respectively) (Supplementary Table S1). No thermal TS were aborted by participants.

### Main effects analysis for quality control

Most main effects (for both groups and sleep conditions combined) were significant, reflecting sufficient quality of our protocol. We found significant adaptation (negative slope for pain in the 5–30 s period, *p* < 0.001, Table 4) and temporal summation of pain (positive slope for pain in the 30–115 s period, *p* < 0.001, Table 4). There were significant CPM effects on thermal mean pain (*p* < 0.001), adaptation (*p* = 0.002), initial peak pain (*p* < 0.001), and PPT (*p* = 0.003, Supplementary Figure S1, Table 4).

**Table 4:** Pressure pain, tonic thermal pain, pain adaption, temporal summation of pain and CPM-effects. Statistical results from the primary analysis

**A: Tonic thermal stimulation**	**Mean coefficient [95% CI] (VAS-pain (cm)/time (seconds))**	***p*-value**
**Adaptation of pain (5–30 s)**		
Main effect (adaptation)	-0.052 [-0.071, -0.032]	*p* < 0.001*
Two-way interaction (migraine, sleep restriction)	0.060 [-0.006. 0.125]	*p* = 0.074
Main effect (CPM)	-0.031 [-0.051, -0.011]	*p* = 0.002*
Three-way interaction (migraine, sleep restriction, CPM)	-0.046 [-0.120, 0.030]	*p* = 0.22
**Temporal summation of pain (30–115 s)**		
Main effect (temporal summation)	0.016 [0.010, 0.022]	*p* < 0.001*
Two-way interaction (migraine, sleep restriction)	-0.012 [-0.031, 0.007]	*p* = 0.23
Main effect (CPM)	-0.003 [-0.008, 0.014]	*p* = 0.34
Three-way interaction (migraine, sleep restriction, CPM)	0.024 [0.003, 0.045]	*p* = 0.023*
Two-way interaction (migraine or sleep restriction and CPM)		
Sleep restriction vs habitual sleep in controls	-0.009 [-0.023, 0.005]	*p* = 0.20
Sleep restriction vs habitual sleep in patients with migraine	0.015 [-0.001, 0.031]	*p* = 0.060
Patients with migraine vs controls after habitual sleep	-0.015 [-0.030, 0.001]	*p* = 0.061
Patients with migraine vs control after sleep restriction	0.009 [-0.006, 0.025]	*p* = 0.24
**B: Tonic thermal stimulation and pressure pain**	**Mean coefficient [95% CI] (VAS-pain in cm or force in N)**	
**Initial peak (5 s) (VAS in cm)**		
Main effect (CPM)	-2.203 [-2.646, -1.762]	*p* < 0.001*
Three-way interaction (migraine, sleep restriction, CPM)	-0.224 [-1.479, 1.030]	*p* = 0.73
**Mean VAS (thermal test stimulus) (VAS in cm)**		
Main effect (CPM)	-2.092 [-2.560, -1.624]	*P* < 0.001*
Three-way interaction (migraine, sleep restriction, CPM)	0.175 [-1.367, 1.716]	*p* = 0.82
**Pressure pain threshold (PPT) (LnN)**		
Main effect (CPM)	0.061 [0.020, 0.102]	*p* = 0.003*
Three-way interaction (migraine, sleep restriction, CPM)	0.041 [-0.122, 0.203]	*p* = 0.62
**Pressure at pain = 5/10 cm (PP5**^**−0.2**^**) (N)**		
Main effect (CPM)	-0.003 [-0.007, 0.000]	*p* = 0.065
Three-way interaction (migraine, sleep restriction, CPM)	-0.001 [-0.011, 0.010]	*p* = 0.92

*CI* Confidence interval, *VAS* Visual Analogue Scale (0–10 cm) for pain recording, *CPM* Conditioned pain modulation, *PPT* Pressure pain threshold. Ln: natural logarithm, *N* Newton, *PP5* Pressure at VAS = 5/10 cm. Contrasts of average marginal effects with 95% confidence interval between base categories (control, habitual sleep, test stimulus (thermal or mechanical)) and alternative category (migraine patient, sleep restriction, CPM) in the primary analysis. A) Contrasts show difference in regression coefficients (VAS-pain (cm)/time (seconds)) for adaptation and temporal summation. B) Contrasts show difference in VAS (cm) for thermal measures or force (N) for mechanical measures. Absolute force (using a 1 cm^2^ probe, 10 N correspond to 100 kilopascal (kPa)). PP5 was calculated based on a regression model between force and pain. PP5 was power transformed, and PPT was log transformed. Coefficients presented for PPT and PP5 are transformed and should be interpreted as such. Due to the power transformation, a negative coefficient for PP5 reflects an increase in PP5 after conditioning stimulus, i.e., a CPM effect. For PPT, a positive coefficient reflects an increase in PPT after conditioning stimulus, i.e., a CPM effect. *p-value < 0.05

### Primary analyses

There was a significant three-way interaction between group (migraine vs. controls), sleep condition (sleep restriction vs habitual sleep), and CPM effect (CS + TS vs TS) on temporal summation of pain (*p* = 0.023, Fig. 4, Table 4). In patients with migraine compared to controls, CPM tended to be decreased after sleep restriction (increased temporal summation of pain during CS) (*p* = 0.060, Fig. 4, Table 4). For the habitual sleep condition, we found the opposite; CPM tended to be increased (decreased temporal summation of pain during CS) in patients with migraine compared to controls (*p* = 0.061, Fig. 4, Table 4). There was no significant interaction between group, sleep, and stimulus condition for adaptation (*p* = 0.22), initial peak pain (*p* = 0.40), thermal mean pain (*p* = 0.85), PPT (*p* = 0.62) or PP5 (*p* = 0.80). For comparison with the piecewise regression model, grand means and individual plots from the thermal part of the CPM protocol can be found in Fig. 3A and B, respectively.

### Secondary and exploratory analyses

**Fig. 5:**
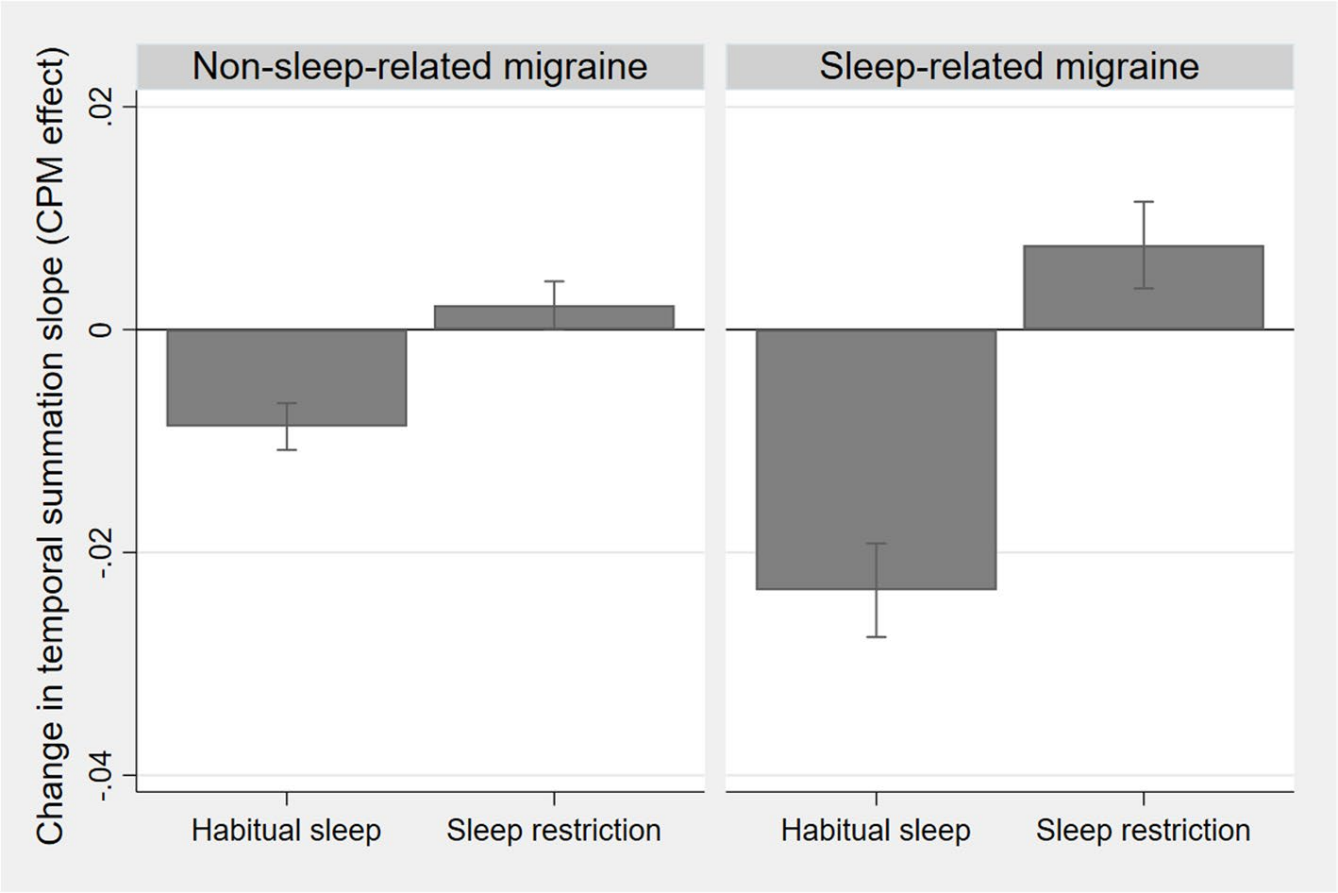
Contrasts from exploratory analyses. CPM: Conditioned pain modulation. Bar plots showing CPM effect on temporal summation of pain for migraine subgroups after habitual sleep and sleep restriction; the bars show change in slope of the temporal summation regression line (ratio of vas/cm) from 30 s with only TS and TS in addition to CS (CPM condition). CPM effect on temporal summation was decreased more after sleep restriction in sleep-related patients with migraine compared to non-sleep-related patients with migraine

Using the 48-h cut-off for the interictal-preictal phase border for the secondary sensitivity analysis, the three-way interaction was confirmed as significant (*p* = 0.016), while the CPM effect after sleep restriction now was significantly decreased in patients with migraine compared to controls (*p* = 0.040, Supplementary Table S5). In the exploratory subgroup analysis, using a 24-h cutoff for the preictal phase, the CPM effect decreased more after sleep restriction (increased temporal summation during CS) in sleep-related migraine compared to non-sleep-related migraine (*p* = 0.017, Fig. 5, Supplementary Table S5).

## Discussion

### Main findings

Our main finding was that for temporal summation of pain, patients with migraine tended to have reduced CPM after sleep restriction between attacks compared to controls. A predefined exploratory subgroup analysis indicated that this effect may be more prominent in patients with sleep-related migraine. These findings indicate that migraine patients, especially those with sleep-related migraine, have decreased endogenous pain modulation following insufficient sleep. We found no differences between patients with migraine and controls when assessing other pain measurements than the CPM-effect on temporal summation.

Some [27, 32, 62], but not all [16, 28, 33–35, 63] studies have found reduced CPM in patients with migraine between attacks. Differing results on CPM in migraine could be related to methodological differences, as stimulus used to elicit temporal summation, and modality of test stimulus and conditioning stimulus in CPM protocols vary considerably in previous studies. Of the studies using a comparable design as in our study, i.e., using tonic heat as and CS, two found similar CPM effect in migraine patients and controls [33, 64]. However, these studies only measured tonic heat for 30 s, and would not capture the temporal summation phase that likely starts between 25 and 65 s using tonic heat (Fig. 3) [65–67]. Additionally, only three other studies reported use of proper blinding procedures [29, 30, 68] where one of these studies found reduced CPM in migraine patients [29]. However, these studies used nociceptive reflexes as TS, making them less comparable to our study. CPM likely reflect the net sum of descending pain pathways [16], as well as involving several cortical areas [20, 63]. We did not find reduced CPM effect following habitual sleep in patients with migraine. However, CPM is reduced in patients with migraine after repeated testing [38], and patients with migraine may have subtle changes in endogenous pain modulation between attacks, representing a subclinical allodynia state [12]. Our findings suggest that subtle changes in endogenous pain modulation in patients with migraine may be provoked by a pain enhancing stressor such as sleep restriction.

Migraine pathophysiology and normal sleep physiology share several neuroanatomical and neurotransmitter pathways [4, 69]. For instance, serotonin is involved in arousal [70], CPM [17], and possibly the hyperalgesic effect of sleep deprivation in rats [71, 72]. Patients with migraine may have some dysfunction in serotonergic pathways between attacks [73, 74], and be vulnerable to decreased serotonin [75]. Speculatively, vulnerability to alterations in serotonergic pathways may explain decreased CPM after sleep restriction in patients with migraine.

Another neurotransmitter involved in sleep and pain modulation is dopamine [70, 76]. Dopaminergic mechanisms are affected differently in patients with migraine and healthy controls by sleep restriction [77], Sleep deprivation reduces activity in dopamine 2- and 3-receptors in the striatum [78], in the nucleus accumbens in the resting state [79], and during tonic pain [80]. Nucleus accumbens may mediate a pronociceptive effect of sleep deprivation [81], and might have decreased volume in patients with migraine [82]. Dopamine may be involved in CPM although evidence is too scarce to conclude [22, 83]. Interestingly, dopaminergic symptoms such as yawning [84], correlate with allodynia during migraine attacks [22, 83–85]. Mykland et al. [7] found that shortening of the cortical silent period, reflecting cortical inhibitory and dopaminergic function [86, 87], in patients with migraine after sleep restriction was associated with allodynia during attacks. Hence, there may be a link between dopaminergic activity and central sensitisation after sleep restriction in migraine.

CPM was significantly reduced after sleep restriction in patients with migraine compared to controls when using a 48-h cut-off for the interictal-preictal phase border. We have used a 24-h cut-off in previous studies, as preictal symptoms are largely specific to the last 24 h [88]. However, preictal symptoms might be present up to 72 h before an attack, and a 48-h cut-off has recently been recommended [89]. We observed a possible effect toward increased pressure pain sensitivity using a 48-h cut-off, and not a 24-h cut-off, in the same participants as in this study [9]. Uglem et al. [39] found increased preictal pain sensitivity, and increased pain sensitivity closer to migraine attacks when omitting preictal measurements. Changes in endogenous pain inhibitory or facilitatory mechanism, may decrease pain sensitivity early in the preictal phase, before increasing closer to the attack [39, 40, 42]. Hence, omitting early preictal measurements may result in a more representative interictal phase, explaining why CPM was significantly reduced after sleep restriction in patients with migraine using a 48-h-cut-off, and not a 24-h cut-off for the interictal-preictal phase border. However, effect sizes were identical (Table 4 and Supplementary Table S5), suggesting that our main CPM result did not depend much on the choice of cut-off for the interictal-preictal phase border.

We found significant CPM effects on thermal mean VAS-pain, adaptation, and PPT when aggregating groups and sleep conditions, suggesting that the CPM protocol was largely successful. We could not confirm a CPM effect on PP5, but the lack of statistical significance could be explained by high variation in measurements (Supplementary Figure S1). Mean VAS-pain change, as a measure of CPM, may be obscured by possible differences in initial peak pain and adaptation between groups and participants (Fig. 3B), whereas temporal summation likely reflects a comparatively more specific measure of endogenous pain modulation. Similar to findings of Tousignant-Laflamme et al. [67] in healthy controls, we could not find a significant main CPM effect on temporal summation of pain, although there were differences between patients with migraine between attacks and controls using this measure. Differences between patients with migraine and controls after sleep restriction could obscure main effects in an aggregated group. We found a significant CPM effect on initial peak pain, in similarity with Tousignant-Laflamme et al. [67]. Initial peak pain and adaptation reflect primarily peripheral mechanisms [67, 90] and the decreased CPM effect in migraine after sleep restriction patients might be specific to more centrally based mechanisms.

### Sleep-related migraine vs non-sleep-related migraine

Differences in endogenous pain modulation between clinical subgroups of migraine may explain varying results in previous studies. In an exploratory analysis, we found that patients with sleep-related migraine had a larger reduction in CPM after sleep restriction (measured by temporal summation of pain during CS) compared with non-sleep-related migraine. Sleep restriction increased thermal pain sensitivity more in sleep-related patients with migraine in a study using the same participants [9]. Non-sleep-related patients with migraine might be relatively sleep-deprived compared to patients with sleep-related migraine, possibly explaining higher pain sensitivity in the non-sleep-related patients with migraine [46]. Endogenous pain inhibition may already be reduced in non-sleep-related patients with migraine at baseline, and the less pronounced pro-nociceptive effect of sleep loss in this subgroup might be explained by a physiological ceiling effect [9]. REM hypoarousability was found in sleep-related patients with migraine compared to controls [91]. This may suggest dysfunctions in serotonergic pathways [74], and the hypothalamus and brainstem [92], as these structures are involved in both migraine and sleep physiology [4]. Dysfunctional serotonergic pathways could render sleep-related patients with migraine more susceptible to sleep restriction.

### Clinical implications and suggestions for future research

There is accumulating evidence toward neurophysiological differences between subgroups of migraine defined by clinical traits, such as sleep-related and non-sleep-related migraine [9, 43, 77]. Clinical traits may be used to predict treatment responses in migraine, as interictal allodynia correlated with poor treatment response to calcitonin gene-related peptide (CGRP) monoclonal antibodies [92]. More knowledge about neurophysiological differences between migraine subgroups and which mechanisms are reflected by CPM may help in choosing between different therapeutic strategies. For instance, reduced CPM predicted more efficacious use of the serotonin-noradrenaline reuptake inhibitor Duloxetine in patients with migraine [33], which could be relevant to a migraine subgroup with reduced CPM after sleep restriction. Treatment of sleep disturbance and focus on sleep hygiene may be more relevant for migraine subgroups, such as sleep-related migraine, that are more vulnerable to the effect of sleep restriction. Future studies on sleep and migraine should consider comparing sleep-related and non-sleep-related migraine patients, to increase knowledge about this potential subgroup.

### Strengths and limitations

One strength of our study is blinding of researchers to sleep condition and diagnosis. The researcher performing the investigations were not involved in recruitment and inclusion of subjects, and we made a large effort to ensure that baseline days and examination days took place in the same way regardless of diagnosis and sleep condition. Blinding procedures in migraine has received attention [93], and reduces risk of biased results [94]. A minority of CPM studies are blinded to patient and control groups, including in migraine studies, and this is a major challenge when comparing results [95]. Another challenge in CPM research is greatly varying methodology, complicating comparisons between studies, as reliability vary with methodology and stimulation parameters [96]. Hence, there is a need for adherence to standardised methods [16]. We mostly complied with methodological recommendations by Yarnitsky [97]. Contrarily, where Yarnitsky [97] recommends painfulness of NRS = 4 for the TS, we used a thermal TS with painfulness of NRS = 6 [54, 55, 98, 99], as higher intensity of TS may decrease the risk of potential floor effects [14]. Additionally, tonic noxious heat stimuli to the volar forearm with painfulness of NRS = 6 as TS and cold-water immersion of the hand has satisfying test–retest reliability [100]. We did not correct for multiple comparisons, and as such there is a possibility of increased likeliness of type I errors. We argue that our findings indicate that the CPM-effect may be reduced in migraine following sleep-restriction, despite that the two-way interaction between CPM-effect and sleep restriction in migraine only was a trend (*p* = 0.06). This interpretation is in line with recent recommendations [60, 100], but we also recognise that the interpretation of *p*-values is challenging and debated [59]. A greater proportion of migraine patients aborted CS compared to controls. This could result in a stronger CPM effect in the control group, because the CPM effect is likely dependent on pain intensity [14], even though it is sustained after CS [101] and is known to last for several minutes following CPM protocols with a CS duration of more than 30 s [102]. However, as the number of aborted CS were similar in migraine patients after sleep restriction and habitual sleep, the number of aborted CS is unlikely to affect the tendency towards reduced CPM in migraine patients after sleep restriction. The sleep-related migraine group was small (*n* = 10). We used the Kenward-Roger correction for small sample inference to alleviate the problem of small subgroups. This subgroup analysis was predefined but followed a non-significant primary analysis, and results should be viewed as preliminary and interpreted with caution. We expected few males to participate due to the higher prevalence of migraine in females [103], and did not define a sex-comparison à priori. We did not ensure that examination occurred at the same phase of the menstruation cycle in female participants. This limitation is unlikely to reflect the result, as the order of sleep conditions were randomised and there were little differences in days from last menstruation in the different groups. The number of oral contraceptive users was imbalanced between sleep-related and non-sleep-related migraine. Non-oral contraceptive users may have reduced CPM effect compared to oral contraceptive users [104]. However, this imbalance is less likely to affect the change in CPM-effect before and after sleep restriction, which is the main outcome in this study.

## Conclusions

In this blinded paired crossover study, CPM tended to decrease after two nights of partial sleep restriction in patients with migraine between attacks compared to controls. Sleep restriction’s effect on CPM may be more pronounced in sleep-related migraine. In conjunction with previous findings, our findings suggest that patients with migraine may have a subtly altered endogenous pain modulatory system that is more susceptible to a pro-nociceptive stressor such as sleep restriction. Experimental sleep restriction may be used to reveal possible subtle neurophysiological differences in patients with migraine between attacks and may be considered in future neurophysiological studies in the interictal phase of migraine.

## Supplementary Information

Supplementary Material 1.

## Data Availability

The datasets used and/or analysed during the current study are available from the corresponding author on reasonable request.

## Abbreviations

CPM: Conditioned pain modulation
DNIC: Diffuse noxious inhibitory control
KSS: Karolinska Sleepiness Scale
ESS: Epworth Sleepiness Scale
ISS: Insomnia Severity Score
PVT: Psychomotor vigilance test
TS: Test stimulus
VAS: Visual analogue scale
NRS: Numerical rating scale
HPTT: Heat pain tolerance threshold
kPa: Kilopascal
N: Newton
PPT: Pressure pain threshold
PP5: Force yielding VAS = 5/10 cm
CS: Conditioning stimulus
SD: Standard deviation
CGRP: Calcitonin gene-related peptide
