# Supplementary material for: Endogenous pain modulation after sleep restriction in migraine: a blinded crossover study
Source: J Headache Pain. 2024 Oct 3;25(1):166. doi: 10.1186/s10194-024-01879-z (PMC11448287; doi:10.1186/s10194-024-01879-z)
Supplement: Supplementary file 1 — Supplementary Material 1. [file 10194_2024_1879_MOESM1_ESM.docx]

# Supplementary materials

# 1.1 Detailed determination of pain6

Based on heat pain tolerance threshold (HPTT), we calculated a tentative pain6 (mean HPTT – 2.5 °C): Three trains of 10 second stimuli were delivered two cm proximal to the flexor groove of the hand to assess whether the tentative pain6 produced a pain intensity of 6 rated on a numerical rating scale (NRS). At the end of the 10 second stimulus, participants scored the pain intensity using a verbal NRS. If the NRS score was different from a pain intensity of 6 on the first 10 second stimuli, the temperature was increased or decreased by 0.5-1 °C determined by the magnitude of the difference from tentative pain6 [1]. This was done three times, producing a corrected pain6, used for the two-minute thermal test stimulus (TS9. We aimed to keep the pain rated on a visual analogue scale (VAS) between two and eight for the thermal TS. If pain intensity was consistently perceived at an intensity below or equal to two or above or equal to nine on the computerised VAS during the first 20 seconds of the thermal TS, the thermal TS was aborted, and pain6 altered accordingly. This was allowed up to five times. Results from the process of determining pain6 can be found in Supplementary Table S1, including mean VAS for each sleep and stimulus condition and number of measurements with VAS outside the 2-8 VAS range. For pain6-temperature, 12 measurements from six test subjects (four migraine patients; two after habitual sleep, two after sleep restriction, two controls; one after habitual sleep, one after sleep restriction), were excluded from the descriptives due to a minor technical issue in the thermal test in either or both test days. In these cases, the thermotest displayed slightly wrong temperatures due to a temporary malfunction that was fixed later. As measurements used for analyses are subjective responses to individually adjusted temperatures, these measurements were still included in analyses.

| **Supplementary Table S1.** | | | | | | | | | | |
| --- | --- | --- | --- | --- | --- | --- | --- | --- | --- | --- |
| Selected pain and performance variables from the conditioned pain modulation (CPM) protocol. | | | | | | | | | | |
|  | | **Controls** | | | |  | **Migraine patients** | | | |
|  |  | **Habitual sleep** | | **Sleep restriction** | |  | **Habitual sleep** | | **Sleep restriction** | |
| Tentative pain6 (°C) |  | 46.0 (2.2) | | 45.9 (2.4) | |  | 45.1 (2.1) | | 45.0 (2.3) | |
| Corrected pain6 (°C) |  | 45.9 (1.6) | | 45.7 (2.0) | |  | 45.2 (1.6) | | 45.3 (1.7) | |
| Final pain6 (°C) |  | 46.2 (1.6) | | 46.0 (1.9) | |  | 45.9 (1.3) | | 45.8 (1.5) | |
| Corrections pain6 (n) |  | 0.8 (1.1) | | 0.8 (1.0) | |  | 1.5 (1.4) | | 1.0 (1.3) | |
| No corrections (n) |  | 17 | | 13 | |  | 8 | | 15 | |
| > 3 corrections (n) |  | 1 | | 1 | |  | 3 | | 3 | |
| Aborted CS (n) |  | 10 | | 4 | |  | 12 | | 14 | |
| Mean NRS-pain (CS)^a^ |  | 8.0 (1.8) | | 8.0 (1.4) | |  | 8.1 (1.6) | | 8.1 (1.6) | |
| Tolerance time, aborted CS (s) |  | 38.6 (7.9) | | 37.5 (8.7) | |  | 35 (19.6) | | 41.7 (13.2) | |
|  |  | **TS** | **CS** | **TS** | **CS** |  | **TS** | **CS** | **TS** | **CS** |
| Mean VAS-pain (thermal TS) |  | 6.3 (1.8) | 4.7 (2.2) | 6.4 (2.0) | 4.7 (2.0) |  | 6.8 (2.6) | 4.3 (2.4) | 6.1 (2.1) | 3.6 (2.4) |
| < 2 mean VAS (n) |  | 0 | 4 | 1 | 3 |  | 1 | 5 | 1 | 10 |
| > 8 mean VAS (n) |  | 5 | 1 | 7 | 3 |  | 12 | 2 | 5 | 3 |
| CS: Conditioning stimulus (right hand immersed in cold water (7 °C)). NRS: Numeric rating scale. VAS: Visual analogue scale (0-10 cm). TS: Test stimulus. Mean (SD) for chosen descriptives from the conditioned pain modulation (CPM) protocol, and descriptives for steps in achieving pain6 used in the thermal test stimulus; this process was repeated for each participant each examination day. ‘Tentative pain6’ was defined as heat pain tolerance threshold - 2.5 °C. The temperature corresponding to Tentative pain6 was applied to the volar forearm three times, in trains of 10 seconds, assessing whether NRS = 6 was consistently reached at the end of the 10 second stimulus. If NRS = 6 was not reached, the temperature was altered correspondingly, producing the ‘Corrected pain6’ used in the CPM experiment. Some participants required further correction of pain6 during the tonic heat stimulation; this was allowed up to five times, producing a ‘Final pain6’. 12 measurements from six test subjects (four migraine patients; two after habitual sleep, two after sleep restriction, two controls; one after habitual sleep, one after sleep restriction), were excluded from these descriptives due to a minor technical issue in thermal test in either or both test days. In these cases, the thermotest displayed slightly wrong temperatures due to a temporary malfunction. As measurements used for analyses are subjective responses to individually adjusted temperatures, these measurements were still included in analyses. . ^a^Participants rated painfulness of the CS by verbal NRS at the end of the two-minute period. The mean values presented are for measurements where the CS was aborted during the CPM protocol. | | | | | | | | | | |

| Supplementary Table S2. | | | | | | | |
| --- | --- | --- | --- | --- | --- | --- | --- |
| Random effects estimates and covariances in the primary analyses. | | | | | | | |
|  | | |  | **Mean coefficient [95% CI] (VAS/time)** | | | |
|  | | |  | **Thermal CPM** | **Mean VAS** | **PPT (LnN)** | **PP5 (N^-0.2^)** |
| Random effects | | |  |  |  |  |  |
|  | Level 2: subject (intercept) | |  | 2.910 [2.134, 3.968] | 1.888 [1.136, 3.135] | 0.190 [0.133, 0.272] | 0.002 [0.001, 0.003] |
|  | Level 2: sleep condition (coefficient) | |  | 1.429 [0.936, 2.182] |  | 0.023 [0.010, 0.053] | 0.000 [0.000, 0.000] |
|  | Level 2: stimulus condition (test stimulus or CPM, coefficient) | |  | 3.000 [2.114, 4.258] | 1.308 [0.529, 3.236] |  | 0.000 [0.000, 0.000] |
|  | Level 2: VAS, 5-30 seconds (adaptation, coefficient) | |  | 0.005 [0.003, 0.007] |  |  |  |
|  | Level 2: VAS, 30-115 seconds (temporal summation, coefficient) | |  | 0.001 [0.000, 0.001] |  |  |  |
|  | Covariance | |  |  |  |  |  |
|  |  | Sleep condition, stimulus condition |  | 0.770 [0.175, 1.364] |  |  | -0.000 [-0.000, 0.000] |
|  |  | Sleep condition, adaptation |  | -0.014 [-0.038, 0.011] |  |  |  |
|  |  | Sleep condition, temporal summation |  | -0.001 [-0.11, 0.008] |  |  |  |
|  |  | Sleep condition, subject |  | -0.814 [-1.381, -0.248] |  |  | -0.000 [-0.000, .000] |
|  |  | Stimulus condition, adaptation |  | -0.027 [-0.052, -0.001] |  |  |  |
|  |  | Stimulus condition, temporal summation |  | -0.04 [-0.013, 0.006] |  |  |  |
|  |  | Stimulus condition, subject |  | -0.837 [-1,487, -0.187] |  |  | -0.000 [-0.000, 0.000] |
|  |  | Adaptation, temporal summation |  | -0.001 [-0.001, -0.000] |  |  |  |
|  |  | Adaptation, subject |  | -0.067 [-0.102, -0.031] |  |  |  |
|  |  | Temporal summation, subject |  | 0.015 [0.004, 0.026] |  |  |  |
|  | Residual | |  | 0.909 [0.747, 1.107] | 2.280 [1.765, 2.946] | 0.053 [0.045, 0.061] | 0.000 [0.000, 0.000] |
| CI: Confidence interval. VAS: Visual Analogue Scale (0-10 cm). CPM: Conditioned pain modulation. PPT: Pressure pain threshold. Ln: Natural logarithm. N: Newton. PP5: Pressure at VAS= 5/10 cm. PP5 was power transformed, while PPT was log transformed, to improve normality of residuals. Coefficients for PPT and PP5 are transformed and should be interpreted as such. Using a 1 cm^2^ probe, 10 N correspond to 100 kilopascal (kPa). PP5 was calculated based on a linear regression model between force and pain. The constant refers to mean VAS for the control group for the habitual sleep condition. Random effects are presented as variances. Random parameters and covariance matrices were included based on likelihood ratio (LR) tests. The random effects and covariances listed in each column are all included in the indicated main model. Values listed under ‘covariance’ are covariances of every perturbation of the five listed random effects. Hypotheses of interest for each main model were extracted from the main model as contrasts. Blank cells indicate that the corresponding random effect was not included in the main model. Hence, there are also no covariances between these values. We used an unstructured variance-covariance matrix, maximum likelihood estimation (MLE), and sandwich estimator for the thermal CPM analyses. We used restricted maximum likelihood estimation (REML) for mean VAS, PPT, and PP5 analyses. | | | | | | | |

| Supplementary Table S3. | | | | | |
| --- | --- | --- | --- | --- | --- |
| Counts from the primary analysis (interictal phase defined by a 24-hour cut-off), secondary sensitivity analysis (48-hour cut-off), and the exploratory subgroup analysis (sleep-related vs non-sleep-related migraine). | | | | | |
|  | |  | **Subjects** | **Examination days** | |
|  | |  |  | **Habitual sleep** | **Sleep restriction** |
| Controls | |  | 31 | 31 | 31 |
| Migraine patients in preictal phase | |  |  |  |  |
|  | 24-hour cut-off |  | 39 | 30 | 30 |
|  | 48-hour cut-off |  | 39 | 30 | 25 |
| Sleep-related vs non-sleep-related migraine^a^ | |  |  |  |  |
|  | Sleep-related migraine |  | 10 | 6 | 7 |
|  | Non-sleep-related migraine |  | 29 | 24 | 23 |

^a^Only performed for 24-hour cut-off

| Supplementary Table S4. | | | | | |
| --- | --- | --- | --- | --- | --- |
| Random effects estimates and covariances in sensitivity analysis (interictal phase defined by a 48-hour cut-off) and exploratory subgroup analysis (Sleep-related migraine vs non-sleep-related migraine). | | | | | |
|  | | |  | **Estimated variances for random effects [95% CI]** | |
|  | | |  | **48-hour cut-off** | **Sleep-related migraine vs non-sleep-related migraine** |
| Random effects | | |  |  |  |
|  | Level 2: subject (intercept) | |  | 2.929 [2.142, 4.005] | 3.486 [2.154, 5.642] |
|  | Level 2: sleep condition (coefficient) | |  | 1.523 [0.957, 2.425] | 1.602 [0.804, 3.192] |
|  | Level 2: stimulus condition (test stimulus or CPM, coefficient) | |  | 2.963 [2.060, 4.262] | 3.712 [2.349, 5.863] |
|  | Level 2: VAS, 5-30 seconds (adaptation, coefficient) | |  | 0.005 [0.004, 0.007] | 0.007 [0.004, 0.010] |
|  | Level 2: VAS, 30-115 seconds (temporal summation, coefficient) | |  | 0.001 [0.000, 0.001] | 0.001 [0.001, 0.010] |
|  | Covariance | |  |  |  |
|  |  | Sleep condition, stimulus condition |  | 0.795 [0.131, 1.459] | 0.980 [-0.053, 2.012] |
|  |  | Sleep condition, adaptation |  | -0.014 [-0.040, 0.012] | 0.003 [-0.041, 0.046] |
|  |  | Sleep condition, temporal summation |  | -0.000 [-0.0100, 0.009] | 0.005 [-.010, 0.020] |
|  |  | Sleep condition, subject |  | -0.877 [-1.494, -0.260] | -1.161 [-2.274, -0.048] |
|  |  | Stimulus condition, adaptation |  | -0.030 [-0.056, -0.005] | -0.049 [-0.102, 0.004] |
|  |  | Stimulus condition, temporal summation |  | -0.003 [-0.012, 0.007] | 0.003 [-0.015, 0.022] |
|  |  | Stimulus condition, subject |  | -0.712 [-1.383, -0.040] | -0.740 [-1.965, 0.485] |
|  |  | Adaptation, temporal summation |  | -0.001 [-0.001, -0.000] | -0.001 [-0.002, 0.000] |
|  |  | Adaptation, subject |  | -0.069 [-0.105, -0.033] | -0.089 [-0.147, -0.030] |
|  |  | Temporal summation, subject |  | 0.015 [0.004, 0.026] | 0.020 [0.001, 0.040] |
|  | Residual | |  | 0.907 [0.741, 1.111] | 0.868 [0.847, 0.889] |
| CI: Confidence interval. VAS: Visual Analogue Scale (0-10 cm). CPM: Conditioned pain modulation. Random effects are presented as variances. Random parameters and covariance matrices were included based on likelihood ratio (LR) tests. Random parameters and covariance matrices were included based on likelihood ratio (LR) tests. The random effects and covariances listed in each column are all included in the indicated main model. Values listed under ‘covariance’ are covariances of every perturbation of the five listed random effects. Hypotheses of interest for each main model were extracted from the main model as contrasts. We used an unstructured variance-covariance matrix, maximum likelihood estimation (MLE), and sandwich estimator for these analyses. | | | | | |

# Supplementary Figure S1. Box plot of pressure pain threshold (PPT) and pressure pain at VAS = 5/10 cm (PP5)


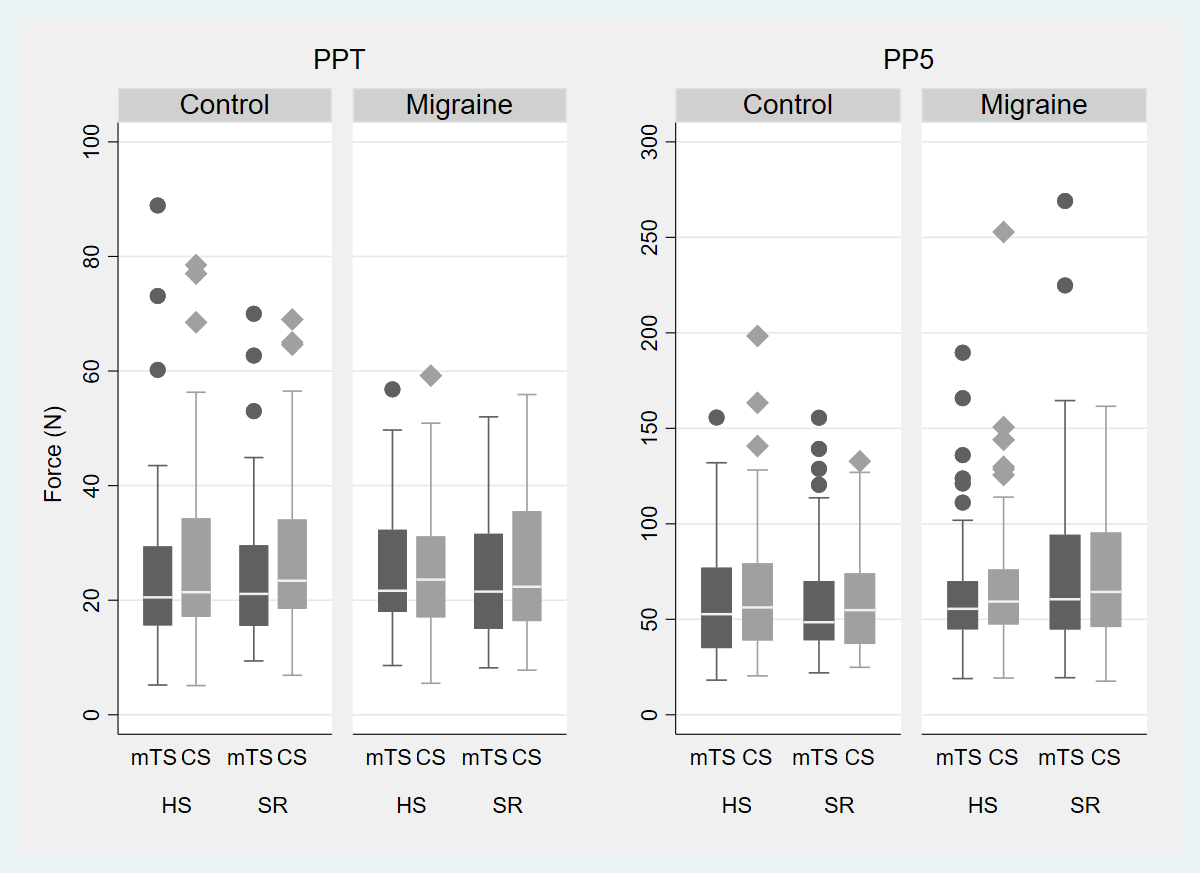


PPT: Pressure pain threshold (newton (N). PP5: Measured pressure pain (N) at VAS = 5/10 cm. mTS: mechanical test stimulus. CS: conditioning stimulus. HS: Habitual sleep. SR: Sleep restriction. Box plot for PPT and PP5 for each combination of group, sleep condition, and stimulus condition. The box plot shows 25th and 75th percentile as borders, and median as a white line. Using a 1 cm^2^ probe, 10 N correspond to 100 kilopascals (kPa). There was a significant CPM effect on PPT (p = 0.003), but not on PP5 (p = 0.065), when combining groups (migraine patients and controls) and sleep conditions (sleep restriction and habitual sleep).

| Supplementary Table S5. | | | | | | |
| --- | --- | --- | --- | --- | --- | --- |
| **A)** Results from the secondary sensitivity analysis (interictal phase defined by a 48-hour cut-off) | | | | | | |
| **Temporal summation of pain (30-115 seconds)** | | | |  | **Mean coefficient [95% CI] (VAS (cm)/time (seconds))** | **p-value** |
|  | Three-way interaction (migraine, sleep restriction, CPM) | | |  | 0.027 [0.005, 0.048] | p = 0.016* |
|  | Two-way interaction | | |  |  |  |
|  |  | Sleep restriction vs habitual sleep in controls | |  | -0.009 [-0.023, 0.050] | p = 0.20 |
|  |  | Sleep restriction vs habitual sleep in migraine patients | |  | 0.018 [0.001, 0.034] | p = 0.040* |
|  |  | Migraine patients vs control after habitual sleep | |  | -0.014 [-0.029, 0.001] | p = 0.08 |
|  |  | Migraine patients vs control after sleep restriction | |  | 0.012 [-0.004, 0.030] | p = 0.14 |
| **B)** Results from the exploratory subgroup analysis in sleep-related and non-sleep-related migraine. | | | | | | |
| **Temporal summation of pain (30-115 seconds) 24-hour cut-off** | | | |  | **Mean coefficient [95% CI] (VAS (cm)/time (seconds))** | **p-value** |
|  | Three-way interaction (SM, sleep restriction, CPM) | | |  | 0.020 [0.014, 0.027] | p < 0.001* |
|  | Two-way interactions | | |  |  |  |
|  |  | | Sleep restriction vs habitual sleep in NSM |  | 0.011 [0.008, 0.014] | p < 0.001* |
|  |  | | Sleep restriction vs habitual sleep in SM |  | 0.031 [0.025, 0.037] | p < 0.001* |
|  |  | | SM vs NSM after habitual sleep |  | -0.015 [-0.019, -0.010] | p < 0.001* |
|  |  | | SM vs NSM after sleep restriction |  | 0.005 [0.001, 0.010] | p = 0.017* |
| CI: Confidence interval. VAS: Visual Analogue Scale (0-10 cm). CPM: Conditioned pain modulation. Contrasts of average marginal effects with 95 % confidence interval between A) patients with migraine between attacks and controls using a 48-hour cut-off for the interictal-preictal phase border, and B) sleep-related and non-sleep-related migraine. Contrasts show difference in regression coefficients for temporal summation. *p-value < 0.05. CI: Confidence interval. | | | | | | |

# 2. Data analysis and statistical analysis

## 2.1. Correcting the rest interval in actigraphy

Rest intervals were automatically defined by the actigraphy software. We corrected rest intervals from the two days preceding each test day semi-manually in a hierarchal manner, with use of the software generated rest intervals, actigraphy event marker for ‘lights off’ and ’lights on’, ‘lights off’ and ‘lights on’ from sleep diary, and light and activity levels from actigraph, respectively [2].

## 2.2. Model specifications

We specified a random intercept for subjects for all analyses. For the piecewise regression model in the thermal part of the CPM protocol, we also specified random coefficients for sleep condition, stimulus condition, and time (5-30 seconds, and 30-115 seconds), and an unstructured variance-covariance matrix. For the mean VAS analysis, we also specified a random coefficient for stimulus condition. For the PPT analysis, we also specified a random coefficient for sleep condition. For the PP5 analyses, we also specified random coefficients for sleep and stimulus condition. We specified an unstructured variance-covariance matrix for the piecewise regression model and PP5. The model for the secondary analysis using a 48-hour cut-off for the interictal-preictal phase border had the same specifications as the primary piecewise regression model.

In cases of less than satisfactory normal distribution of residuals, we compared standard errors (SE) with and without using the sandwich estimator (robust estimator of variance) to assess possible model misspecification [3]. The piecewise regression model in the primary analyses had less than satisfactory normal distribution of residuals and discrepancy between SEs with and without sandwich estimator. Hence, maximum-likelihood estimation (MLE) with sandwich estimator was used for the piecewise model as restricted maximum-likelihood estimation (REML) is incompatible with the sandwich estimator. REML was used for all other analyses as REML is less biased for balanced data compared to MLE [4].

For the exploratory analyses in sleep-related and non-sleep-related migraine, we specified a random intercept for subject, random coefficient for sleep condition, stimulus condition, and time (5-30 seconds, and 30-115 seconds). This analysis had similar specifications as the primary analyses, as two-level models with recordings nested in subjects, and a fixed part including main effects of the migraine subgroup, sleep condition, and stimulus condition (CPM vs thermal TS), and their respective three-way interaction and two-way interactions. Random coefficients and covariance matrices were included based on likelihood ratio tests. We used an unstructured variance-covariance matrix, and restricted maximum likelihood estimation (REML), but not sandwich estimator for analyses on non-sleep-related vs sleep-related migraine as the Kenward-Roger correction for small sample inference is incompatible with the sandwich estimator.

# References

1. Matre D, Andersen MR, Knardahl S, Nilsen KB: **Conditioned pain modulation is not decreased after partial sleep restriction**. *Eur J Pain* 2016, **20**(3):408-416.

2. Follesø HS, Austad SB, Olsen A, Saksvik-Lehouillier I: **The development, inter-rater agreement and performance of a hierarchical procedure for setting the rest-interval in actigraphy data**. *Sleep Medicine* 2021, **85**:221-229.

3. King G, Roberts ME: **How Robust Standard Errors Expose Methodological Problems They Do Not Fix, and What to Do About It**. *Political Analysis* 2015, **23**(2):159-179.

4. Rabe-Hesketh S, Skrondal A: **Multilevel and Longitudinal Modeling Using Stata, Volumes I and II, Third Edition: Multilevel and Longitudinal Modeling Using Stata, Volume II ... Counts, and Survival, Third Edition**: Stata Press; 2012.
